# Fatal Multisystem Inflammatory Syndrome in Adult after SARS-CoV-2 Natural Infection and COVID-19 Vaccination

**DOI:** 10.3201/eid2711.211612

**Published:** 2021-11

**Authors:** Heather N. Grome, Michael Threlkeld, Steve Threlkeld, Charles Newman, Roosecelis Brasil Martines, Sarah Reagan-Steiner, Michael A. Whitt, Maria Gomes-Solecki, Nisha Nair, Mary-Margaret Fill, Timothy F. Jones, William Schaffner, John Dunn

**Affiliations:** Centers for Disease Control and Prevention, Atlanta, Georgia, USA (H.N. Grome, R.B. Martines, S. Reagan-Steiner);; Tennessee Department of Health, Nashville, Tennessee, USA (H.N. Grome, M.-M. Fill, T.F. Jones, J. Dunn);; Baptist Memorial Health Care, Memphis, Tennessee, USA (M. Threlkeld, S. Threlkeld); Methodist LeBonheur Healthcare, Memphis (M. Threlkeld, S. Threlkeld);; Pathology Group of the MidSouth, Germantown, Tennessee, USA (C. Newman); Trumbull Laboratories, Germantown (C. Newman);; University of Tennessee Health Science Center, Memphis (M.A. Whitt, M. Gomes-Solecki, N. Nair);; Vanderbilt University School of Medicine, Nashville (W. Schaffner)

**Keywords:** COVID-19, coronavirus disease, SARS-CoV-2, severe acute respiratory syndrome coronavirus 2, viruses, respiratory infections, multisystem inflammatory syndrome in adults, MIS-A, vaccines, vaccine-preventable diseases

## Abstract

We describe a fatal case of multisystem inflammatory syndrome in an adult with onset 22 days after a second dose of mRNA coronavirus disease vaccine. Serologic and clinical findings indicated severe acute respiratory syndrome coronavirus 2 infection occurred before vaccination. The immunopathology of this syndrome, regardless of vaccination status, remains poorly understood.

A multisystem inflammatory syndrome in children (MIS-C) and adults (MIS-A) occurring after coronavirus disease (COVID-19) has been identified; onset is ≈4–6 weeks after severe acute respiratory syndrome coronavirus 2 (SARS-CoV-2) infection (*1*–*3*). A case definition for MIS-A has been developed by the Centers for Disease Control and Prevention (CDC) (*4*); MIS-A after vaccination is rare and remains of great clinical and public health interest (*5*). We report a case study and histopathologic findings from a fatal MIS-A case after SARS-CoV-2 infection and subsequent complete COVID-19 vaccination.

## The Patient

The patient was a healthcare worker in his 30s with no notable medical history. In December 2020, he experienced mild COVID-19–like illness symptoms, including fatigue and loss of taste and smell. He did not undergo testing for SARS-CoV-2 at that time and was unaware of the need for isolation. Six days after onset of COVID-19–like symptoms, and when fully recovered, the patient received the first dose of Pfizer/BioNTech (https://www.pfizer.com) mRNA COVID-19 vaccine. He received the second dose 20 days later. After the second dose, he reported fatigue and malaise, which resolved within 2 days.

Twenty-two days after receiving the second dose of the COVID-19 vaccine, he had onset of new fever, malaise, headache, and odynophagia. He was examined by an outpatient medical provider. Diagnostic testing was notable for a negative COVID-19 test by reverse transcription PCR (RT-PCR), negative rapid influenza antigen, and negative rapid antigen detection for group A *Streptococcus.* Four days later, the patient visited an emergency department because of worsening symptoms. Assessment of vital signs revealed a temperature of 37.2°C, heart rate 113 beats/min, and blood pressure of 117/66 mmHg. Physical examination identified right-sided cervical lymphadenopathy, marked bilateral conjunctival erythema, and a faint papular rash on the pelvis and left flank. Laboratory testing revealed a peripheral-blood leukocyte count of 11,000 cells/μL, 93.5% segmented neutrophils, and thrombocytopenia with a platelet count of 110,000/μL (Table). Portable chest radiograph results were without notable findings.

**Table:** Results of pertinent laboratory testing completed during the 4-day hospitalization of a patient with fatal multisystem inflammatory syndrome in adult, Tennessee, USA, 2021*

Variable	Hospital day				Reference range
	Day 1	Day 2	Day 3†	Day 4†	
Hematologic testing					
Peripheral leukocyte count, 1,000/μL	11.0	14.4	28.1	8.8	4.2–10.2
Hemoglobin, g/dL	13.7	11.7	10.8	9.5	12.8–16.4
Hematocrit, %	39.6	33.8	34.3	27.7	38.8–48.1
Platelets, 1,000/μL	110.0	86.0	45.0	17.0	150–400
Absolute neutrophils, 1,000/μL	10.5	13.7	23.9		1.8–7.1
Absolute lymphocytes, 1,000/μL	0.4	0.4	5.0		1.3–5.9
Segmented neutrophil, %	95.3	95.3	76.6		40.0–76.0
Lymphocytes, %	3.8	2.8	15.9		14.0–46.0
Monocytes, %	1.9	0.9	0.9		4.0–12.0
Chemical testing					
Creatinine, mg/dL	1.2	1.3	3.4	1.9	0.70–1.30
AST, U/L		78.0	5,938.0	8,861.0	15–37
ALT, U/L		61.0	3,386.0	3,421.0	16–61
Total bilirubin, mg/dL	1.9	1.4	1.6		0.2–1.0
Alkaline phosphatase, U/L	76.0	74.0	295.0	202.0	45–117
Ferritin, serum, mg/dL		1434.9	>40,000.0		26.0–388.0
Coagulation					
aPTT, s	29.3		54.2	80.9	23.2–34.1
PT, s	13.9		39.6		11.7–14.5
INR	1.1		4.2	3.6	0.9–1.0
Fibrinogen, mg/dL	642.0		750.0		208–475
D-Dimer, µg FEU/mL	5.2	4.3	12.2		0.0–0.44
Cardiac					
Troponin-I, ng/mL		18.0	15.5		0.0–0.045
Immunochemical testing					
ESR, mm/h	40.0				0–15
C-reactive protein, mg/L		284.0	174.0		<3.0
Procalcitonin level, ng/mL		4.9			0.50–2.0
Microbiologic testing					
SARS-CoV-2 RT-PCR, index value	Negative				Negative
SARS-CoV-2 IgG antibody,‡ index value	4.96				<1.39
Adenovirus DNA PCR, qualitative	Not detected				Not detected
CMV PCR, quantitative	Negative				Negative
Mononucleosis screen	Negative				Negative
*Ehrlichia chaffeensis* DNA PCR	Not detected				Not detected
HIV-1 p24 Ag	Nonreactive				Nonreactive
Peripheral blood culture, 2 sets	No growth	No growth	No growth	No growth	No growth

*Laboratory values represent pertinent laboratory results during the patient’s hospitalization. Not all laboratory studies completed during hospitalization are represented in this table. Blank cells indicate test not done. ALT, alanine aminotransferase; AST, aspartate aminotransferase; aPTT, activated partial thromboplastin time; CMV, cytomegalovirus; ESR, erythrocyte sedimentation rate; FEU, fibrinogen equivalent units; INR, international normalized ratio; PT: prothrombin time; RT-PCR, reverse transcription PCR; SARS-CoV-2, severe acute respiratory syndrome coronavirus 2. †Laboratory values indicate studies after cardiac arrest, which occurred at ≈3 a.m. on hospital day 3. Note patient was initiated on extracorporeal membrane oxygenation shortly after return of spontaneous circulation; anticoagulation treatments affect laboratory values. ‡SARS-CoV-2 IgG test specific for nucleocapsid protein antibody.

On hospital day 2, the patient remained febrile and tachycardic (heart rate 90–135 beats/min) and had a blood pressure of 92/56 mmHg. Diagnostic evaluation revealed a negative SARS-CoV-2 RT-PCR test but a positive serologic test for SARS-CoV-2 nucleocapsid IgG. Additional diagnostic tests were conducted (Table). Inflammatory markers showed elevated C-reactive protein at 284.0 mg/L, serum ferritin at 1434.9 ng/mL, and troponin-I at 18.0 ng/mL. On the evening of hospital day 2, the patient received 75 g of intravenous immune globulin (IVIG).

Early morning on hospital day 3, the patient had an acute change in mental status, including confusion and global aphasia. An emergent computed tomography scan of the head was negative for cerebrovascular accident and showed normal brain parenchyma and no evidence of acute infarction, mass, or hemorrhage. On completion of the scan, the patient was found nonresponsive and without a pulse. He underwent multiple rounds of advanced cardiac life support, resulting in return of spontaneous circulation. A chest radiograph showed an enlarged cardiac silhouette, and an echocardiogram showed severe biventricular dysfunction, severe global hypokinesis of the left ventricle, and left ventricular ejection fraction of 20%. The patient received a second dose of IVIg and intravenous steroids and extracorporeal membrane oxygenation support was initiated. On hospital day 4, severe multisystem organ failure continued to progress. The patient died on hospital day 4.

We reviewed the patient’s medical history and clinical chart. We assessed serum samples collected during the hospital course before and after IVIg, and we determined endpoint titers to SARS-CoV-2 nucleocapsid (IgM and IgG) and spike receptor binding domain with neutralization functions against spike protein (*6*,*7*). The endpoint titer was a modified protocol based on Stadlbauer et al. (*8*). We completed an autopsy and sent formalin-fixed, paraffin-embedded tissues to CDC. Microscopic examination of lung, airways, pulmonary lymph node, liver, heart, spleen, kidneys and stomach tissue samples was performed; LT-Gram stain was performed on lungs and heart. An RT-PCR assay for SARS-CoV-2 was performed on RNA extracted from formalin-fixed, paraffin-embedded tissues from lungs, airways, and heart by methods previously published (*9*). This activity was reviewed by CDC and was conducted consistent with applicable federal law and CDC policy.

Serum antibody results drawn before IVIg infusion were negative for SARS-CoV-2 IgM but positive for IgG. Serum results had a high titer of anti-spike receptor binding domain antibody both before and after IVIg (1:75,000) compared with a naturally infected SARS-CoV-2–positive control (1:4,000). In addition, the pre-IVIG sample serum results demonstrated neutralizing function.

Notable findings on gross internal autopsy examination included a 525-mL pericardial effusion and cardiac enlargement, as well as a 5-L hemoperitoneum and a 20-cm diameter perisplenic hematoma. Microscopic examination of the lungs showed diffuse congestion, increased intra-alveolar macrophages, multifocal hemorrhage, capillaritis, and microthrombi throughout (Figure, panels A, B). We observed no viral inclusions or diffuse alveolar damage. Trachea and bronchi showed mild tracheobronchitis. Sections of the heart showed multifocal myocarditis with mixed inflammatory infiltrate, myocyte necrosis, and numerous microthrombi. We also identified disseminated microvascular thrombosis in the heart, stomach, kidneys, and liver (Figure, panels C–F). Gram stain results were negative on lung and heart tissue. SARS-CoV-2 RT-PCR was negative on lungs, trachea, bronchi, and heart.

**Figure:**
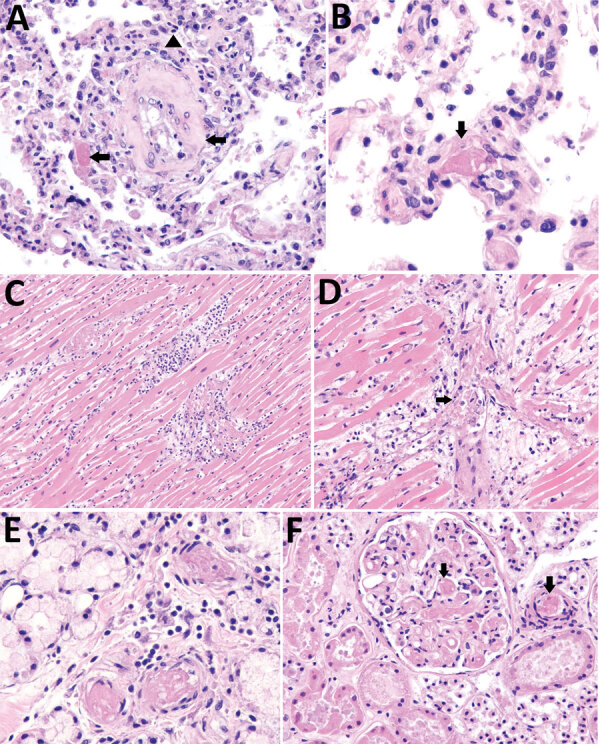
Histopathologic findings in a fatal case of multisystem inflammatory syndrome in adult after natural severe acute respiratory syndrome coronavirus 2 infection and coronavirus disease vaccination, Tennessee, USA, 2021. A) Lung tissue shows capillaritis characterized by neutrophilic inflammation and necrosis within interalveolar septa (arrowhead). Fibrin and organizing intraluminal microthrombi in small arteries are also seen (arrows). Original magnification 20×. B) Higher magnification of fibrin microthrombus within a lung vessel (arrow). Original magnification 63×. C) Heart tissue shows myocarditis with myocyte necrosis and mixed inflammatory infiltrate. Original magnification 20×. D) Higher magnification cardiac vessel showing microthrombus and perivascular mononuclear inflammatory infiltrate (arrow). Original magnification 40×. E) Stomach tissue shows submucosal microthrombi with perivascular lymphocytic infiltrate. Original magnification 63×. F) Kidney tissue shows multiple fibrin thrombi in glomerular (arrow) and interstitial capillaries (arrow). Original magnification 40×.

## Conclusions

This fatal case of MIS-A occurred after full COVID-19 vaccination in a patient with prior natural SARS-CoV-2 infection suspected 6 weeks before MIS-A symptom onset. Serum antibody results before IVIg infusion indicated the patient was previously infected with SARS-CoV-2 and was vaccinated with a COVID-19 vaccine. Antibodies to the nucleocapsid protein are the most sensitive target for serologic diagnosis for natural infection (P.D. Burbelo et al., unpub. data, https://doi.org/10.1101/2020.04.20.20071423), and these antibodies are not present following COVID-19 vaccination alone. In addition, clinical history was compatible with natural infection beginning 6 days before the first mRNA vaccine dose and consistent with negative SARS-CoV-2 nucleocapsid IgM on testing during hospitalization.

The patient demonstrated similar clinical findings to previously reported MIS-A cases, including fever for 3 consecutive days, laboratory evidence of inflammation, neurologic and mucocutaneous clinical findings, and severe cardiac illness that included systemic hypotension progressing to cardiogenic shock (*1*,*5*). These criteria meet the CDC case definition for MIS-A, as well as a definitive case at level 1 of diagnostic certainty by the Brighton collaboration case definition for MIS-A and MIS-C (*10*). In addition, the histopathologic findings of capillaritis and multiorgan microvascular thrombosis in association with clinical symptoms and laboratory findings are compatible with MIS-A (*1*,*11*). Substantial blood loss on gross examination may represent a diffuse intravascular coagulation–type picture in which diffuse microthrombosis depleted platelets and clotting factors. The etiology for clinical deterioration was likely multifactorial, although considerable cardiac compromise in the setting of high fluid volumes and intraperitoneal hemorrhage may have contributed to multiorgan failure

Whether mRNA COVID-19 vaccination contributed to MIS-A onset in this case is unclear, and future epidemiologic studies are needed to understand whether an association exists. The immunopathology leading to hyperinflammation causing MIS-A after SARS-CoV-2 infection remains unknown, although postinfection immune dysregulation is consistent among reported cases. Notably, MIS-A has not been reported among adult participants of COVID-19 vaccine trials (*10*), and no direct evidence exists to support vaccine alone as the primary etiology in this case. This article further emphasizes the importance of COVID-19 prevention, for which infection prevention strategies and vaccination remain our greatest defense.
